# Just about Right

**Published:** 1859-09

**Authors:** 


### JUST ABOUT RIGHT.

About a hundred years ago, a merchant's son concluded to marry. He had knocked around the world a good deal, had lived in boarding houses, and by looking and thinking, had learned something of every day domestic life. One of the lessons was, that quite a large number of the jarrings of the married state of some, arose from circumstances of the most trivial character, in consequence of each party wanting to have its own way. So with mercantile exactness, he entered into an agreement with his affianced, that when they were married, in all matters in which a difference of opinion arose, his voice should rule. "Henceforth their lives were not two, but one. Their minds were nicely fitted to each other. They worked harmoniously together." This gentleman was a doctor theoretically, for he took more delight in the study of medicine than anything else. By what means he was led to devise this "compound" cannot now be known, but, like most valuable medicines, it certainly would be "hard to take," a most unwelcome dose to modern belles, even with the "bonus" of a large fortune, and social position. But a bargain is a bargain with all honest minds, and in this case, it was faithfully adhered to by both parties, and the historian relates—"The union was perfectly happy." The union of Henrietta Leeds, with John Howard, of immortal memory.

